# Awareness about breast cancer and mammogram among women attending outpatient clinics, Ain Shams University Hospitals, Egypt

**DOI:** 10.1186/s42506-019-0026-5

**Published:** 2019-12-04

**Authors:** Ayat F. Manzour, Dina A. Gamal Eldin

**Affiliations:** Department of Community, Environmental and Occupational Medicine, Faculty of Medicine, Ain Shams University, Cairo, Egypt

**Keywords:** awareness, breast cancer, screening, mammogram

## Abstract

**Background:**

Breast diseases in women, whether benign or malignant, are very commonly encountered. The breast is the commonest site for female cancer in Egypt (38.8%). Breast cancer screening can reduce morbidity and mortality and improve the survival rate for this malignancy. Mammogram can be used as a screening technique beside its role as diagnostic, especially in women over 40 years of age.

**Objectives:**

To assess knowledge, attitude and practice regarding breast cancer and awareness about mammogram as a screening method among a group of females attending Ain Shams University outpatient clinics.

**Methods:**

A cross-sectional study was performed from August to September 2017. A systematic random sample was taken from attending females (18–70 years) in Ain Shams University outpatient clinics (Internal Medicine, Surgery, Pediatrics Hospital, and Maternity hospital). They were interviewed using a questionnaire inquiring about sociodemographic background, participants’ knowledge, attitude, and practice towards breast cancer and its screening.

**Results:**

The mean age ± SD of attending females (18–70 years) was 37 ± 11 years. Most study participants had correct information about mammography. They showed poor knowledge level about risk factors. Mass media such as TV and internet were identified as the main source of information on breast cancer by 43% and 23.9%, respectively. In general, participants had positive attitude towards breast cancer screening by mammography. Around 90% agreed that mammogram was the best way to find a very small lump in the breast, and 91.4% agreed that women who have regular screening by mammogram have better disease outcome than those who do not screen. Regarding mammography practice rate, a small percent of participants (8.1%) was advised by their doctors to conduct a screening mammography. The level of knowledge was significantly and positively correlated with their attitude towards breast cancer screening.

**Conclusion:**

The poor knowledge and practices of women illustrate the need for health education program directed to Egyptian females to improve their knowledge about breast cancer—especially its risk factors—and its screening. Using TV and Internet as media for spreading information about this disease is crucial.

## Introduction

Breast cancer is the most frequently diagnosed cancer among women which now represents one in four of all cancers in women. It is also the most common cause of death among women worldwide [1].

Recently, there is a shift toward Western lifestyles in developing countries due to rapid societal and economic changes. These styles consequently result in dietary, reproductive, and hormonal changes which are risk factors for the dramatic increase in cancer rates. Although, the incidence rates of breast cancer are higher in developed countries, mortality is much greater in developing countries, due to difficulty in early detection of the disease and lack of access to treatment as well [2]**.**

Globally, breast cancer is the most diagnosed cancer and the leading cause of cancer death among females, representing 23% of the total cancer cases and 14% of the cancer deaths. Breast cancer is now also the leading cause of death among women from all cancers in developing countries [3]. Additionally, breast cancer mortality rates in African women are higher in comparison to women living in Western countries [4]**.**

In Egypt, the age adjusted rate of breast cancer is 49.6 per 100.000 population and the median age for diagnosis is one decade younger than European countries and most female patients are pre-menopausal [5]**.** Breast cancer in young age is generally more aggressive and may result in lower survival rates, so early detection is very important and even more crucial to raise breast cancer awareness among young females [6]**.**

Several factors are known to affect the risk for the development of breast cancer. Age, family history, and reproductive factors are the strongest risk factors. Lifestyle and hormonal risk factors have also been mentioned [7]**.**

Knowledge of risk factors of breast cancer for females and perception of their personal risk are the most important factors for motivation of females for the prevention, early detection, and management of the disease [8]. On the other hand, lack of knowledge about breast cancer and its risk factors has also been identified as an important factor which can prevent women from participating in screening for breast cancer [9]**.**

The most common and used breast cancer screening methods in the world in order are self-examination, clinical breast examination, and mammography [10, 11]**.** Mammography is currently the only recommended imaging method for breast cancer screening. The American Cancer Society recommends for all females from the start of age of 40 mammograms to be conducted every year and to be continued as long as a woman is in good health. In addition, clinical breast exam (CBE) is recommended every year for women aged 40 and more. All major US medical organizations recommend screening mammography for women aged 40 years and older. Screening mammography is very useful as it reduces mortality from breast cancer by about 20–35% in women aged 50–69 years and slightly less in women aged 40–49 years at a period of 14 years of follow-up [12]**.**

Mammogram is well known as a screening method, but to be effective, it needs that women should have adequate knowledge and attitude towards mammogram when used as a screening method. The present study aimed to assess knowledge, attitude, and practice regarding breast cancer and mammogram as a screening method among a group of females attending Ain Shams University outpatient clinics.

## Participants and methods

### Study design, setting, and population

A cross-sectional study was performed in Ain Shams University teaching hospital, outpatient clinics (including internal medicine, pediatrics, and general surgery) during August and September 2017. Attending females (18–70 years) were interviewed using a questionnaire developed by Amin, 2008 [13]**.**

### Sample size

The sample size was calculated based on expected knowledge regarding breast cancer, and its screening was 50%, *α* error = 0.05, and power = 0.8, and accordingly, the sample size was calculated to be 384 participants. A total of 384 were interviewed of whom 3 participants did not wish to the complete questionnaire. The sample size was calculated using power and sample size program version 11 [14].

### Sampling technique

A list of patient names in the outpatient clinics (internal medicine, pediatrics, and general surgery) was obtained in the morning. A systematic random sample was used; every third patient was interviewed.

### Study tool

An interview questionnaire derived from Amin [13] was used. It was tested through a pilot study of 10 women. Data from the pilot study was not included in the final results**.** The internal consistency was (*r* = 0.74). Two questions were removed from the original questionnaire before testing as they were not applicable to our study setting. These were about “Language commonly used at home” and “Degree of fluency in English.” The study by Amin [13] was done on migrants. A third question was also removed as it annoyed all pilot study participants. The question was “Does pain caused by tight bra lead to breast cancer?”

The questionnaire included (a) 14 background questions; age, marital status, educational level, occupation, residence, number of children, monthly expenditure, presence of family physician, and if the participant reached menopause or not. Participating women were asked to rate the most important channel—from their point of view—of health education to deliver information about breast cancer and its screening (TV, radio, Internet, etc.). (b) Participants’ knowledge about breast cancer epidemiology, and its screening was tested through 12 questions (most common type of female cancer, overweight and other risk factors, Egypt is one of the top Arab countries regarding prevalence of breast cancer, most common age for developing cancer, etc.). An answer was scored “one” for correct answer and “zero” for wrong answer or “Don’t know.” Scores of the twelve questions were then summed and treated as a continuous variable. (c) Participants’ attitude was explored through 14 questions (women’s attitude towards screening by mammogram, its effectiveness in discovering very small lumps, finding free time to schedule for mammogram, etc.). A five-point Likert Scale was used to allow the individual to express their attitude towards a particular statement. Scores for the 14 questions were summed and treated as a continuous variable. Participants’ answers ranged from “Strongly agree to strongly disagree.” 1 = strongly disagree, 2 = disagree, 3 = neutral, 4 = agree, while 5 = strongly agree. The range of the total score is 14–70 (14 questions × 1–5). (d) Practice; two questions were asked to participants (If her doctor ever asked her to do a mammogram and if the participant had a mammogram due to any reason).

### Data management

Data was coded, entered, and analyzed using SPSS (Statistical Package For Social Sciences) [15].

### Data analysis

Descriptive statistics was done using the number and percentage for categorical variables, mean ± SD, and range for quantitative variables. Bar chart was used to express the most important sources of information. Pearson’s correlation was used to find relation between women’s knowledge and their attitude towards mammogram as a screening technique. Linear regression was performed to find out factors which independently affect participants’ knowledge. *P* value of < 0.05 was considered statistically significant.

## Results

Our sample consisted of 381 females, aged (18–71) years with mean ± SD (37 ± 11) years. The majority were housewives and residents of Cairo. The most frequent level of education was secondary education (33.1%) followed by “below secondary education” (25.5%). Most of them were married (84.3%) and (90%) reported having children; mean ± SD (3 ± 1) children. Female participants reported starting age of pregnancy at 12 years and kept getting pregnant until 48 years. The majority (81.9%) were in childbearing age (Table 1). Having a family physician was reported by only 10.2%. Slightly more than half (53%) earned 500 to less than 2000 Egyptian pounds monthly while 18.6% earned 2000 pounds or more. The remaining percentage 28.4% earned less than 500 Egyptian pounds monthly.

**Table 1:** Sociodemographic characteristics of study participants, Ain Shams University hospital, outpatient clinics, Egypt, 2017

Characteristics	No.	Percent
Marital status		
Married	321	84
Divorced/widow	28	7.4
Single	33	8.6
Reaching menopause (no)	312	81.9
Level of education		
Illiterate	79	20.7
Below 2ry education	97	25.5
2ry education	126	33.1
University/postgraduate	79	20.7
Occupation		
Housewife	317	83.2
Working	64	16.8
Residence		
Greater Cairo	330	86.6
Other governorates	51	13.4
Having children (yes)	329	90.1
Age 18–70 years, mean ± SD 37 ± 11		
≤ 20 year	10	2.6
21–30 years	119	31.2
31–40 years	131	34.3
41–50 year	75	19.6
51 + years	47	12.3
Number of children (1–10 children) mean ± SD	3 ± 1	
Age of participant at birth of 1st child (12–36 years) mean ± SD	21 ± 4	
Age of participant at birth of the last child (17–48 years) mean ± SD	30 ± 6	

**Fig. 1:**
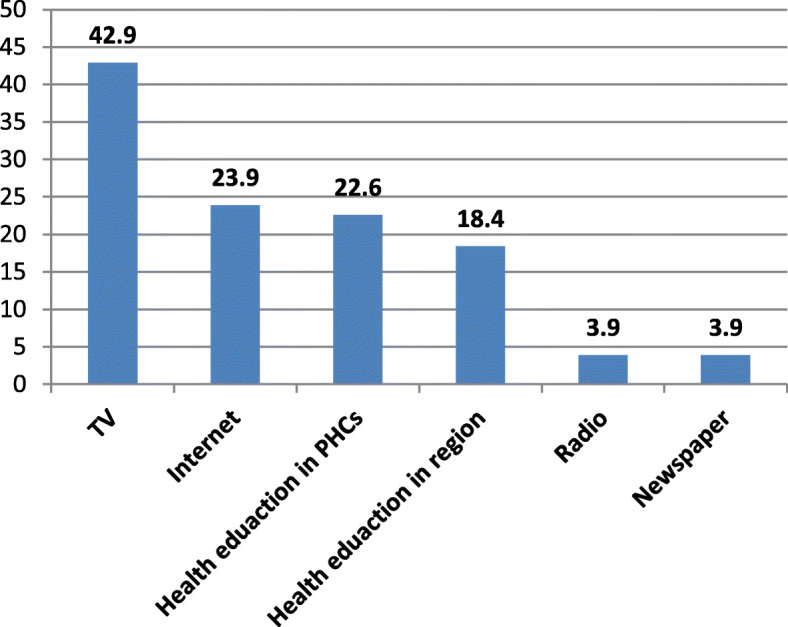
The most common sources of knowledge about breast cancer and its screening (multiple answers were allowed)

The most common sources used by study participants for getting information was TV (42.9%) followed by Internet and the health education received in some primary health care centers (23.9 and 22.6%, respectively). The least used sources were radio and newspapers (3.9% each) (Fig. 1).

Most of study participants had correct knowledge about mammography (detects masses which are undetectable by hand, recommended above 40 years, and improves cure opportunity) (93.2%, 85.5%, and 85.3%, respectively). Regarding knowledge about risk factors, they showed poor knowledge level (overweight is a risk factor, old age at 1st child is a risk factor, probability of breast cancer increases with age, and benign tumors do not increase risk of breast cancer) (40.9%, 32.3%, 26.5%, and 12.9%, respectively) (Table 2).

**Table 2:** Knowledge about breast cancer risk factors and methods of its screening

Knowledge	Correct answer	
	No.	%
Breast masses undetectable by hand can be detected with radiodiagnostic procedures	355	93.2
Mammogram is recommended above 40 years	327	85.8
Radiological examination improves cure opportunity	325	85.3
Clinical examination improves cure opportunity	312	81.9
Breast cancer is the most common cancer in women	267	70.1
Most breast lumps are not cancer	237	62.2
Breast cancer in Egypt is more common than Arab countries	232	60.9
Who discovers breast lumps?	212	55.6
Overweight is a risk factor	156	40.9
Old age at 1st child is a risk factor	123	32.3
Probability of breast cancer increases with age	101	26.5
Benign tumors do not increase risk of breast cancer	49	12.9

In general, participants had positive attitude towards breast cancer screening by mammography. This was evident as 89.9% agreed that having a mammogram is the best way to find a very small lump in the breast, and 91.4% agreed that women who have regular screening by mammogram have better disease outcome than those who do not screen. When participants were asked about details of mammogram procedure, nearly half of them answered “I don’t know” (average time taken to undergo a mammogram, painful or not, and whether it exposes women to unnecessary radiation). More than half (57.4%) disagreed that they do not have enough time to schedule for mammograms; nevertheless (51.4%) agreed that they have more important problems to take care of (Table 3).

**Table 3:** Attitude of participating women towards breast cancer screening

Attitude statement	Disagree/strongly disagree		Undecided		Agree/strongly agree	
	No.	%	No.	%	No.	%
If I get a mammogram and nothing is found, I will not worry as much about breast cancer	70	18.4	44	11.6	266	70
Having a mammogram will help me find breast lumps easily	7	1.9	33	8.7	340	89.4
If I find lumps through my routine mammogram, my treatment for breast cancer may not be as bad as those who do not do regular mammogram	4	1	29	7.6	347	91.4
Having a mammogram is the best way for me to find a small lump in the breast	6	1.5	33	8.7	342	89.8
Having a mammogram will decrease my chances of dying from breast cancer	70	18.4	106	27.8	205	53.8
I am afraid to have a mammogram as I might find out something is wrong	260	68.6	27	7.1	92	24.3
I am afraid of having mammogram as I do not understand what the mammogram procedure involves	201	52.9	35	9.2	144	37.9
I do not know how to do mammogram	170	44.6	126	33.1	85	22.3
Having a mammogram is too embarrassing procedure	241	63.2	11	2.9	129	33.9
Mammogram procedure takes a long time	126	33.1	201	52.8	54	14.1
Having a mammogram is too painful	151	39.6	182	47.8	48	12.6
Having a mammogram exposes me to unnecessary radiation	126	33.1	165	43.3	90	23.6
I do not have enough time to schedule for mammograms	219	57.4	37	9.7	125	32.9
I have other problems that are more important	164	43.1	21	5.5	196	51.4

The knowledge score of study participants ranged between 0 and 11 points (out of 12) with mean ± SD (7 ± 2) while attitude score ranged between 35 and 65 points (out of 70) with mean ± SD 49.4 ± 5.7. Knowledge and attitude score were significantly and positively correlated (*r* = 0.133, *p* = 0.01) (Table 4).

**Table 4:** Correlation between participants’ knowledge and attitude

		Attitude Mean ± SD (32 ± 3.5
Knowledge Mean ± SD (7.1 ± 2)	Pearson correlation	.133
	P value	.010

Regarding participants’ practices of breast cancer screening, only 8.1% were advised by their doctor to have mammogram while 3.4% already performed mammogram (Table 5).

**Table 5:** Practices of participating women towards breast cancer screening

Practices		No.	%
Did your doctor advised you to conduct mammogram of the breast?	Yes	31	8.1
Have you ever done mammogram scans for each breast separately to detect breast cancer?	Yes	13	3.4

Linear regression shows that age and level of women’s education independently affected their level of knowledge (*p* < 0.001 and 0.038, respectively) (Table 6).

**Table 6:** Linear regression model showing effect of some socio-demographic factors on participants’ knowledge

Variables	Coefficients B	t	p	95.0% Confidence Interval for B	
				Lower limit	Upper limit
Age	.046	4.928	<0.001	.027	.064
Education	.903	2.085	.038	.051	1.755
Marital status	-.103	-.367	.714	-.655	.449
Residence	.116	.390	.697	-.469	.702
Occupation	-.115	-.416	.678	-.660	.430
(Constant)	5.359	10.806	<0.0001		

## Discussion

The current study showed that the majority of study participants had low level of knowledge regarding breast cancer risk factors which was disappointing given their exposure to health education sessions and also contact with health workers such as doctors and nurses. This finding agreed with Boulos and Ghali [16] study among Ain Shams University female students aged from 17 to 23 years, who showed that most study participants had low level of knowledge about breast cancer risk factors. More than 85% of study participants knew that mammogram was recommended above the age of 40 which disagrees with Ojewusi and Arulogun’s study, 2016, in their Nigerian study [4]. They found that 1.6% only of their participants knew that 40 years is the recommended age for mammogram. This may be due to the growing awareness and free of charge breast cancer screening campaigns in Egypt. These campaigns together with mass media role may have contributed to improvement of people’s knowledge and attitude.

We found that knowledge about breast cancer risk factors improved significantly with age (*p* < 0.001) which disagrees with Dahiya et al. [17]. They found that Indian women aged < 30 showed significantly higher knowledge scores (*p* < 0.01). This controversy is probably due to that Indian females below 30 years received very good health education sessions in schools and universities as stated by participants. Another cross-sectional study done in Buraidah, Saudi Arabia, on teachers working in female schools [18] using a self-administered questionnaire reported that more than half of participating women showed limited knowledge level (52.1%). Also, in their study older age (40 years and over) who were working in secondary schools had significantly higher knowledge level.

Regarding knowledge about breast cancer risk factors in the current study finding was lower than Boulos and Ghali [16] study as 59.1% of women in the present study did not know that overweight is a risk factor for breast cancer in comparison to nearly 45% of female students at Ain Shams University Hospitals.

More than two thirds of the women (68%) in the current study did not know that old age at first child was another risk factor for breast cancer which agreed with Boulos and Ghali study [16] (57.1% of female students at Ain Shams University Hospitals). On the other hand, according to Dahiya et al.’s study [17], 56.3% of participating women knew that old age at first child was a risk factor for breast cancer.

Most of participating women in this study (73.5%) were not aware that the probability of breast cancer increases with age but Boulos and Ghali [16] study showed that only 33% of female students at Ain Shams University Hospitals were not aware of this fact. This may be because in Boulos and Ghali’s [16] study, they recruited highly educated females from University, while in our study, there was a high percentage of illiterates and below 2ry education participants (46%). On the other hand, 71.4% of Indian women were not aware that breast cancer increases with age and this was according to Dahiya et al. [17]. Lack of awareness about this unmodifiable risk factor was also reported in Ojewusi and Arulogun’s study [4] (79.2%).

As for warning signs of breast cancer, most of participating women in this study (87%) did not know that breast lumps or benign tumors increase the risk for breast cancer. Sixty-two percent were aware that most breast lumps are not cancer, compared to 16.2% of female students at Ain Shams University Hospitals [16]. However, 57% of female respondents knew that breast lump was an essential symptom of breast cancer.

While 74.2% of female students in Boulos and Ghali’s [16] study identified breast self-examination (BSE) as an early detection measure for breast cancer, 82% of women in the current study were aware that clinical breast examination (CBE) improves cure opportunity. This may reflect awareness of study participants of early detection either by self-examination or by clinicians’ hands. Meanwhile, more than half of female students (52.1%) in Ain Shams University identified mammogram as an early detection measure for breast cancer [16]**.** Moreover, in our study, 93% were aware that undetectable breast masses by hand could be detected by radio diagnostic procedures as they are more sensitive. This result agreed with Newton and Grethlein [19] who revealed that the earliest sign of breast cancer can be an abnormality depicted on a mammogram, before it can be felt by the woman or her physician. Sambanje and Mafuvadze in their study in Angola [7] showed that 69.4% and 72.4% of medical and non-medical students, respectively, reported having knowledge of BSE. However, the majority of the students (more than 60%) were not aware that a change in the color or shape of the nipple could be a sign of breast cancer and more than 50% did not know the best time to perform BSE.

In this study, 86% of participants were aware that mammogram was recommended above 40 years and this result was found in a group of women with good knowledge about healthy lifestyle and healthy practices including spacing between births and family planning [20]**.** This result disagrees with Ojewusi and Arulogun’s study [4] in which only 3.3% identified the age of 40 as a recommended age for mammogram screening. According to Milicent et al. [20], women who were most likely to benefit from mammography aged 50 to 69 years, as this age had high prevalence of low breast density. The majority of participants in our study (85.3%) knew that detecting breast lump by radiological method improves treatment opportunity which agrees with Dahiya et al. [17].

Regarding the source of health information and knowledge, 43% of women in this study had their information from mass media (TV) and this agreed with Boulos and Ghali’s [16] study (89.1%) and Allam and Abd El Aziz [5] in Egypt (60%), while the least common source of knowledge in this study and Boulos and Ghali [16] was newspapers. This may be due to the fact that TV is the most readily available method of mass communication and media. It is also easy to understand for illiterates. Conversely, Dandash and AL-Mohaimeed [18] revealed that 83.2% of participants mentioned that newspaper was the most commonly reported source of information about cancer. This was followed by television (68.2 %), family and friends (28.6%), and lastly 14.1% reported their source of information by health care providers. This disagreement is possibly due to cultural difference as some communities depend more on reading for improving their knowledge.

Concerning screening for breast cancer and awareness about mammogram, our study showed that 86% of participants were aware that mammogram was recommended as a screening method for breast cancer above 40 years. The proportion of women who had ever heard of mammography was about 5% in a study conducted by Milicent et al. [20]. The higher proportions reported in the current study is most likely a reflection of the high level of education of the studied sample where 33.3% of the sample had secondary level of education and 21% had university education compared to 32% from Milicent’s study, which was carried out on 818 women attending the General Outpatient clinic (GOP) of the University College Hospital (UCH), Ibadan, Nigeria. Educated women were more likely to benefit from messages regarding breast cancer knowledge and preventive methods and thus were more likely to learn about mammography.

Regarding mammography practicing rates, 91.9% of interviewed women were not advised by their doctors to conduct a screening mammography at any stage of their life and only 8.1% of women were advised to do mammography with 8 women had done it routinely. This finding may be explained by the poor knowledge and skills of health care providers in providing health education and their poor attitude regarding health education. So, clinicians need to be trained on health education skills in order to give information to women on the most current investigation or screening methods as these patients may never get this valuable information from other settings apart from health care settings. Further studies should be done to explore health care workers’ needs in this regard, and provision of training is mandatory. Additionally, the utilization of breast cancer screening services can serve as a true means of obtaining heath information concerning screening methods such as mammography.

In a study conducted among Arabian women living in Qatar, by Donnelly et al. [21], they reported that 24.4% of female participants had talked with their doctors about breast cancer. According to that study, the most common reasons for not performing a mammogram was because their doctors did not recommend it for them (49.7%). However, most of female participants reported the following reasons for performing mammogram: (98.2%) performed mammogram to make sure about their own health, (86.2%) performed it based on their doctor’s recommendation, and (76.3%) performed it for fear of getting cancer.

As regards some beliefs and attitudes related to mammogram practice, 63.2% of interviewed women disagreed that having a mammogram was a very embarrassing procedure and this finding agreed with Donnelly et al. [21] who concluded that 84% of participants had a belief that mammogram is not an embarrassing procedure. This result indicates a positive attitude from females participating in our study and may be due to the campaigns done regularly in the Egyptian mass media.

Despite appreciating the important role of mammogram in early detection, fear of discovering something wrong by the mammogram was the main reason mentioned for not performing the scan (68.6%) rather than being embarrassing (63.2%) or painful (39.3%). This contradicts with the findings of Donnelly et al. [21] where the most common reason for deferring from mammography were possible pains (23%) and fear of cancer being discovered (15.5%). On the other hand, Amin’s study on Arabic speaking women in Nova Scotia, Canada [13], found similar reasons for not performing mammogram.

Women in the current study showed favorable attitude towards the importance of mammogram in early detection of breast lumps as the majority (89.4%) agreed that having a mammogram will help them find breast lumps easily, 89.8% agreed that having a mammogram is the best way for them to find a small lump in the breast while 91.4% agreed that finding lumps through routine mammogram, may improve their treatment for breast cancer. Moreover, 53.8% agreed that having a mammogram will decrease their chances of dying from breast cancer. This result agrees with Donnelly et al. [21], where they found that approximately two thirds had a positive attitude towards screening in general and planned to have a CBE or a mammogram. However, in Dandash and AL-Mohaimeed’s study [18], most of female participants (58.2%) held pessimistic views about the curability of breast cancer and less than one third (29.0%) agreed to perform screening for early detection of breast cancer.

When participants of our study were asked if they have more important problems to manage, 51.4% agreed to this statement. This may be explained by the fact that there is a big percentage of female Egyptians who lead the household (61%) according to a report by United Nations [22]. This poses higher burden on the woman and often results in limited time and resources for monitoring her health status.

The current study results also showed that higher education improves participants’ knowledge which agreed with Gürdal et al. [23] in Turkey who found that higher education was associated with a more common belief in early diagnosis of BC. This is obviously because higher educational level makes people able to find knowledge sources about different diseases and to have positive attitude to their screening. On the other hand, Ojewusi and Arulogun [4] found in their study on Nigerian teachers that higher level of education was not a significant determinant of BSE practice and hence their attitude towards screening. This might be an effect of culture that overcomes effect of education. Worth mentioning despite being highly educated, 27% of participants in Ojewusi and Arulogun’ study [4] thought that breast cancer is caused by devil.

### Study limitations

This study was a hospital-based study and may not truly reflect the level of awareness among the general population. Our sample included only the outpatients from Ain Shams University clinics who were present at time of data collection, so it cannot be generalized to all women of different socio-economic classes.

## Conclusion and recommendations

Exploring awareness of female attendants at Ain Shams University outpatient clinics revealed that those women had good level of knowledge regarding breast cancer epidemiology and its status in Egypt; however, their knowledge regarding the risk factors of this disease was poor and need to be improved so that they can identify high-risk groups. Generally, participants had positive attitude towards performing mammogram for screening of breast cancer; nevertheless, they were overwhelmed with other life problems keeping them away from performing screening. Age and educational level independently affected participants’ knowledge.

This study clearly illustrates the need for health education programs directed to Egyptian females attending Ain Shams University outpatient clinics to improve their knowledge about breast cancer especially its risk factors and its screening by mammogram after 40 years.

There is a crucial need to conduct health education sessions for females early in their childbearing period in schools and universities to raise their awareness about breast cancer and its risk factors. Training of health care professionals (HCPs) to educate women about this serious and potentially fatal type of cancer is a must as HCPs can be a trustable source of information.

## Data Availability

Available on reasonable request.
